# Impact of Fungicides Chlorothalonil and Propiconazole on Microbial Activities in Groundnut (*Arachis hypogaea* L.) Soils

**DOI:** 10.5402/2011/623404

**Published:** 2011-12-25

**Authors:** A. C. Ramudu, G. Jaffer Mohiddin, M. Srinivasulu, M. Madakka, V. Rangaswamy

**Affiliations:** ^1^Department of Microbiology, Sri Krishnadevaraya University, Anantapur 515055, Andhra Pradesh, India; ^2^Department of Biotechnology and Bioinformatics, Yogi Vemana University, Kadapa 516 003, Andhra Pradesh, India

## Abstract

Introduction of agrochemicals (fungicides) into soil may have lasting effects on soil microbial activities and thus affect soil health. In order to determine the changes in microbial activity in a black clay and red sandy loam soils of groundnut (*Arachis hypogaea* L.) cultivated fields, a case study was conducted with propiconazole and chlorothalonil to evaluate its effects on soil enzymes (cellulase and invertase) throughout 40 days of incubation under laboratory conditions with different concentrations (1.0, 2.5, 5.0, 7.5, and 10.0 kg ha^−1^). Individual application of the two fungicides at 1.0, 2.5, and 5.0 kg ha^−1^ to the soil distinctly enhanced the activities of cellulase and invertase but at higher concentrations of 7.5 and 10 kg ha^−1^ was toxic or innocuous to both cellulase and invertase activities. In soil samples receiving 2.5–5.0 kg ha^−1^ of the fungicides, the accumulation of reducing sugar was pronounced more at 20 days, and the activity of the cellulase and invertase was drastically decreased with increasing period of incubation up to 30 and 40 days.

## 1. Introduction

The economy of India is largely dependent on the quality and quantity of agricultural produce. Better harvest requires intensive cultivation, irrigation, fertilizers, and more importantly pesticides to protect plants from pests and plant diseases. Pesticides are one of the widely used products developed by man in the last century. They have a beneficial impact not only on agricultural productivity along with the reduction of costs but also on the quality of life and improvement of longevity. It has been reported globally that about 3 × 10^9^ kg of pesticides are applied annually [1]. In India, 15–20% of agricultural production is negatively influenced by pests [2]. Currently, different fungicides are used to protect crops against fungal plant pathogens in agricultural practices and maintain high crop production in modern agriculture. Groundnut (*Arachis hypogaea* L.) is one of the important major, profitable oil seed crop grown throughout the year in India [3]. Groundnut ranks seventh among crops in terms of insecticide consumption in India. It contributes to 41.3% of countries oil seed production [4]. When a pesticide is released deliberately or accidentally into the environment, about 0.1% is reaching the target organism, while the remaining 0.99% not only troubles local metabolism or enzymatic activities [5–9], but also disturbs soil ecosystem, and thus may affect human health by entering in the food chain, which has raised considerable public concern. So, from past 10 decades, more specific prominence has been given to soil enzymes because these are indicators of biological equilibrium, fertility, quality [10–12] and changes in the biological status of soil due to pollution [13, 14]. When compared with enzymes from different sources, soil enzymes commonly show particular and peculiar feature. Soil enzymes are involved in energy transfer, nutrient cycling, environmental quality, and crop productivity. Negative impact of pesticides on soil enzymes like hydrolases, oxidoreductases, and dehydrogenase activities has been widely reported in the literature [15, 16]. But on cellulase and invertase, too little literature is available on chlorothalonil and propiconazole (fungicides). Cellulase and invertase enzymes are also very important enzymes involved in the transformation/decomposition of organic matter in soil. Cellulase catalyzes hydrolysis of cellulose to D-glucose. Invertase is a ubiquitous enzyme that occurs in plant tissues and soil organisms [17, 18]. Invertase hydrolyze sucrose to fructose and glucose. As previously reviewed by so many researchers, several studies [15, 19–22] have been performed on the impact of pesticides on soil microbial activity, and application of pesticides increased, decreased, or did not affect the activities of these enzymes in soils, depending upon the nature and concentrations of pesticides used, incubation period, status of enzymes in soil, and soil condition. Chlorothalonil (2, 4, 5, 6-tetrachloroisopthalonitrile), a nonsystemic widely used foliar fungicide for the control of plant pathogens causing broad spectrum of plant diseases in agricultural systems. Propiconazole is a systemic foliar fungicide with a broad range of activity. It is used on grasses grown for seed, mushrooms, corn, wild rice, peanuts, almonds, sorghum, oats, pecans, apricots, peaches, nectarines, plums, and prunes. Therefore, investigating effects of contaminants on enzymatic activity may evaluate the soil microbial properties and further prove extremely useful in risk assessment.

## 2. Materials and Methods

### 2.1. Soils Used in the Present Study

Two soils, a black clay soil and red sandy loam soil, were collected randomly from different sites of groundnut cultivated fields of Anantapur district of Andhra Pradesh, India, near the rhizosphere zone (a zone of increased microbial and enzyme activity where soil and root make contact) using trowel at a depth of 0–12 centimeters and mixed thoroughly to prepare a homogenate composite sample, air-dried at room temperature samples were cleaned by removing plant material and other debris, passed through 2 millimeter sieve, stored at 4°C prior to analysis. Mineral matter of soil samples were done by the following methods. Soil pH was determined by using 1 : 1.25 soils to water ratio in systronic digital Ph meter [23]. Organic matter in soil samples was estimated by Walkley-Black method [24], and total nitrogen content in soil samples was determined by Micro-Kjeldhal method [24]. Electrical conductivity was measured by conductivity bridge and contents of nitrite—nitrogen [25] by Brucine method [26]. The important physicochemical properties of the two soils were furnished in Table 1.

### 2.2. Insecticides Used in the Present Study

To determine the influence of selected insecticides on soil enzyme activities, propiconazole (25% emulsifying concentration*), and chlorothalonil (75% wettable powder*) was obtained from Sergeant India Ltd. 14, Tata Road, Mumbai-20. Obtained commercial insecticides were dissolved in distilled water.

### 2.3. Soil Incubation Studies

#### 2.3.1. Cellulase and Invertase Activities

Five-gram portions of soil samples were weighed and dispersed into sterile test tubes (25 × 150 mm). Stock solutions from selected fungicides were added at the rate of 10, 25, 50, 75, and 100 *μ*g g^−1^ soil, which are equivalent to field application rates of 1.0, 2.5, 5.0, 7.5, and 100 kg ha^−1^, respectively. Soil samples without fungicides treatment served as controls. Soil samples were mixed thoroughly for uniform distribution of fungicides added. Triplicates were maintained for each treatment at room temperature (28 ± 4°C) with 60% water holding capacity throughout the incubation period. After desired intervals of incubation, soil samples were extracted in distilled water for estimation of enzyme activities.

### 2.4. Assay of Cellulase (EC 3.2.1.4.)

In order to determine cellulase enzyme activity in soils, 10 mL of carboxy methyl cellulose (CMC) 1% was used as a substrate followed by 10 mL of acetate buffer (pH 5.9) and incubated for 24 hours to determine the reducing sugar content in the filtrate [27]. In another experiment, rate of enzyme activity cellulase were determined at 10, 20, 30, and 40 days of soil incubation and further with the respective suitable substrate.

### 2.5. Assay of Invertase (EC 3.2.1.26)

The method employed for assay of invertase was developed by [28] and followed by Tu [29, 30]. The soil samples were transferred to 100 mL Erlenmeyer flasks and were treated with 1 mL of toluene to arrest the enzyme activity. After 15 minutes, 6 mL of 18 mM sucrose was added to the soil samples and incubated for 24 and 48 hours, the testing samples were passed through Wattman no. 1 filter paper, and the filtrate was assayed for the amount of glucose by Nelson somagi method [31] in a spectronic 20D spectrophotometer. In another experiment, rate of enzyme activitiy invertase were determined at 10, 20, 30, and 40 days of soil incubation and further with the respective suitable substrate.

### 2.6. Statistical Analysis

The concentration of the cellulase and invertase was calculated on the basis of soil weight (oven dried). Data were analyzed using one-way ANOVA and the differences contrasted using Duncan's multiple range test (DMRT) [19, 32]. All statistical analysis was performed at *P* ≤ 0.05 using SPSS statistical software package.

## 3. Results

### 3.1. Cellulase (EC 3.2.1.4.)

**Figure 1:**
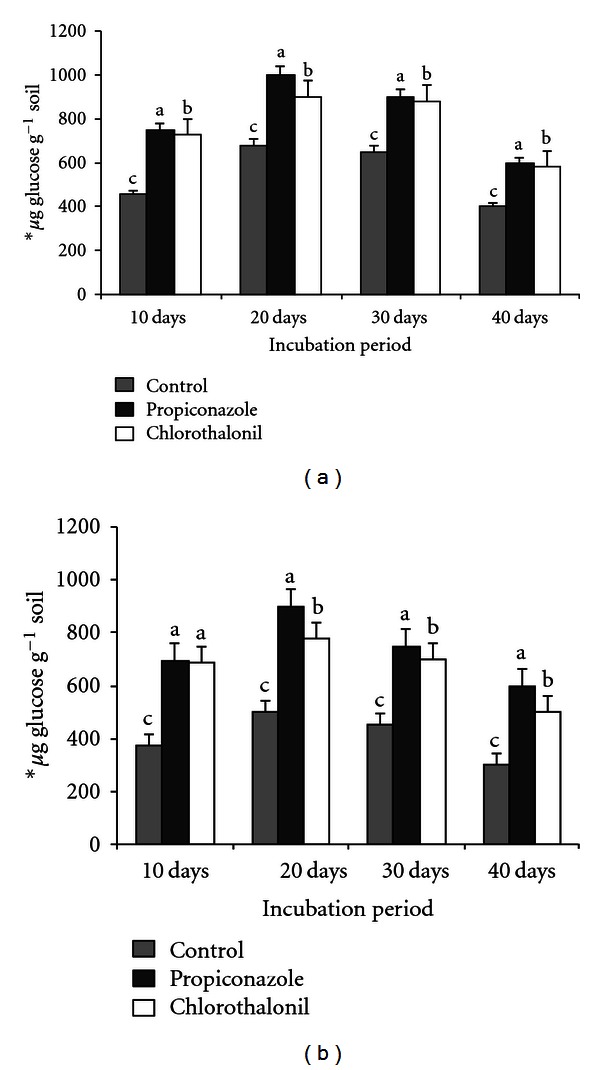
Influence of fungicides at 5.0 kg ha^−1^ on cellulase* activity in black clay soil (a) and red sandy loam soil (b) *μg of glucose per gram soil formed after 24 hours with 1% carboxy methyl cellulose (CMC) after 10, 20, 30, and 40 days. The values are the means ± S.E. for each incubation period, followed by the different letter are significantly different (*P* ≤ 0.05) from each other according to Duncan's multiple range (DMR) test.

The effect of different application rates of chlorothalonil and propiconazole on cellulase activity is presented in Table 2. After 10 days of incubation enzyme activity increased in all the treatments (1.0, 2.5, 5.0, and 7.5 kg ha^−1^) except at 10.0 kg ha^−1^ level. The maximum cellulase activity was observed at 5.0 kg ha^−1^ (stimulatory) and lowest activity at 10.0 kg ha^−1^ level. The cellulase activity was significantly enhanced at 5.0 kg ha^−1^ level in both soils for chlorothalonil and propiconazole showed individual increments of cellulase activity ranged from a low increase 3–59%, 10–83%, 9–65%, and 15–85% in comparison to control (Table 2). The stimulatory concentration (5.0 kg ha^−1^) induces the highest enzymatic activity after 20, 30, and 40 days of incubation in both soils (Figures 1(a) and 1(b)) when compared to control. A further increase in the incubation period of stimulatory concentration of fungicides decreased the rate of cellulase activity after 20 days and then decline phase was started from 20 to 40 days of incubation (Figures 1(a) and 1(b)).

### 3.2. Invertase (EC 3.2.1.26)

**Figure 2:**
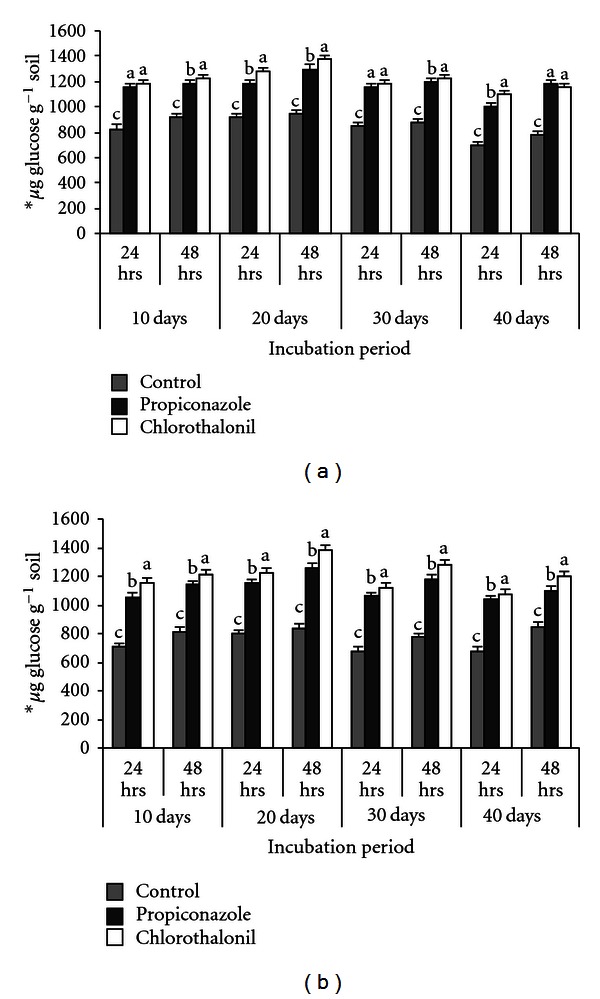
Influence of fungicides at 2.5 kg ha^−1^ on invertase* activity in black clay soil (a) and red sandy loam soil (b) after 24 and 48 hours. *μg glucose per gram soil formed after 24 and 48 hrs incubation with 18 mM sucrose. After 10, 20, 30, and 40 days. The values are the means ± S.E. for each incubation period, followed by the different letter which are significantly different (*P* ≤ 0.05) from each other according to Duncan's multiple range (DMR) test.

Invertase activity (Tables 3(a) and 3(b)) showed a variable pattern in response to different fungicide concentration after 10 days of incubation. Enzyme activity increased under all the treatments (1.0, 2.5, 5.0, and 7.5 kg ha^−1^) except 10 kg ha^−1^ level compared to the controls in both soils. The maximum activity was observed at 5.0 kg ha^−1^ (stimulatory) for chlorothalonil and 2.5 kg ha^−1^ (stimulatory) for propiconazole, lowest activity at 10.0 kg ha^−1^ level for both fungicides in both soils (Tables 3(a) and 3(b)). chlorothalonil and propiconazole showed individual increments of invertase activity ranged from a low increase 8–43%, 21–63% and 10–39%, 7–49% in comparison to control at 24 hrs and for 48 hrs 2–33%, 12–48% and 9–130%, 9–40% received 5.0 and 2.5 kg ha^−1^, respectively (Table 3). The stimulatory concentration induces the highest enzymatic activity after 20, 30, and 40 days of incubation in comparison with control in both soils (Figures 2(a) and 2(b)). With further increase in the incubation periods for a prolonged period (up to 40 days), the stimulatory concentration of fungicides decreased the rate of invertase activity after 30 days, and then, decline phase was started from 30 to 40 days of incubation (Figures 2(a) and 2(b)).

## 4. Discussion

The activity of invertase, as evidenced by the accumulation of glucose formed from sucrose was consistently more than that of cellulase activity in soil samples. In both red sandy loam soil and black clay soil samples, the concentrations ranging from 1.0–5.0 kg ha^−1^ were either stimulatory or innocuous to the enzyme activity. However, treated with 2.5 kg ha^−1^ of tilt and 5.0 kg ha^−1^ of kavach in both soils showed maximum enzyme activity at the end of 24 and 48 hrs of incubation with substrate (i.e., sucrose). Application of these fungicides at 7.5 kg ha^−1^ and 10.0 kg ha^−1^ significantly inhibited the formation of glucose from sucrose. The data presented in Tables 3(a) and 3(b) showed the activity of invertase under the influence of different concentrations (1.0, 2.5, 5.0, 7.5, and 10.0 kg ha^−1^) of fungicides after 10 days of incubation. In all untreated red and black soil samples, invertase activity was significantly more at 20-day incubation when compared with 10-day, 30-day, and 40-day incubated soil samples (Figures 2(a) and 2(b)). All the treated soil samples showed their assayed enzyme activity more in the soil samples of 20-day incubation when compared with 10-days, 30-days, and 40-day incubation (Figures 2(a) and 2(b)). The soil samples (black and red) treated with 2.5 kg ha^−1^ of tilt and 5.0 kg ha^−1^ of kavach, the enzyme activity was more (Figures 2(a) and 2(b)). Further more this increase in glucose concentration was striking when the substrate was exposed to the soil samples for 48 hrs. For minor variations in enzyme activity, both (tilt and kavach) fungicides exhibited stimulatory effect significantly until 20 days. Prolonged incubation (up to 40 days) of fungicide-treated soil samples showed either stimulation or no measurable effect on the enzyme activity. When compared the results with others on the invertase activity [20, 33–36], the results obtained were similar. Comparatively, in the present study, the black soil showed higher enzyme activity than the red soil throughout the experiment. It is usually concluded that high enzymatic activities are associated with higher organic matter content, Tu [33] reported two fungicides, triazophos a phosphoro-thioate triazole and the invertase activity was increased to 10-fold. A similar effect was observed with respect to thiram [34]. On the contrary, captan and maneb, at the same concentrations and incubation period, had no effect on invertase activity [29, 30, 33]. The authors in [37] demonstrated that captafol and chlorothalonil suppressed invertase activity for one day temporarily in a sandy loam soil, and later on, after 2 days, the inhibitory effect diminished. Similarly, Srinivasulu and Rangaswamy [20] reported decrease in the invertase activity at higher concentrations (7.0 and 10.0 kg^−1 ^ha) by the application of tridemorph and captan. The activity of invertase was significantly inhibited by chlorothalonil up to 37.7%, 13.9, and 34.2%, respectively [21]. Yan et al., [38] noticed that, the carbendazim (8.0 mg kg^−1^) and chloramphenicol showed inhibitory effect on invertase activity. A negative correlation was observed between the napropamide and invertase activity by Guo et al., [39]. The outcome of this investigation fairly indicates that the fungicides used in agriculture at levels nearer to field doses significantly enhanced the invertase activity in soil environment.

The method developed and used for the assay of cellulase activity in soils is based on colorimetric determination of reducing sugars in soil extracts formed from the carboxy methylcellulose in the presence of soil cellulase. Glucose formed from carboxy methylcellulose (CMC) was significantly more in both red sandy loam and black clay soil samples treated with 5.0 kg ha^−1^ (Table 2) of propiconazole and chlorothalonil which showed more cellulose activity. Application of fungicides at 7.5 kg ha^−1^ and 10.0 kg ha^−1^ significantly inhibited the formation of glucose from CMC. The data presented in the Table 2 showed the activity of cellulase under influence of different concentrations (1.0, 2.5, 5.0, 7.5, and 10.0 kg ha^−1^) of fungicides after 10 days. Rangaswamy and Venkateswarlu in [22] noticed that the insecticides at higher concentrations of 7.5 and 10.0 kg ha^−1^ were toxic to cellulase activity. Jayamadhuri [35] and Jayamadhuri and Rangaswamy [36] reported that higher concentrations (7.5 and 10 kg ha^−1^) of fungicides reduce the enzyme activity. In all the untreated red sandy loam soil and black clay soil samples, The amount of glucose formed from cellulose was significantly more at 20 day incubation, when compared with 10-days, 30-days, and 40-days incubated samples (Figures 1(a) and 1(b)). Similarly, all the treated soil samples showed their assayed enzyme activity more in the soil samples of 20 days incubation when compared with 10-days, 30-days, and 40-days incubation. As for the soil samples treated with propiconazole and chlorothalonil, enzyme activity was more at concentrations of 5.0 kg ha^−1^. Expect for minor variations in enzyme activity, all the fungicides exhibited stimulatory effect significantly until 20 days after their application. Prolonged incubation (up to 40 days) of fungicides-treated soil samples showed either stimulation or no measurable effect on the enzyme activity. Tu [33, 34], Jayamadhuri [35], and Jayamadhuri and Rangaswamy [36] observed similar trend of cellulase activity. However, high concentrations of 7.5 and 10.0 kg ha^−1^ levels of two fungicidal treatments had innocuous effect on cellulase activity in both soil samples (Table 2). Similarly, an anthraquic fluvisol soil incubated with the formulated fungicide, hymexazol for 4 weeks remained unchanged in cellulolytic activity [40]. From the experimental data, it is clear that stimulatory effect was comparatively more in black clay soil than red sandy loam soil (Table 2). The stimulatory effect on cellulase activity was maximum at 5.0 kg ha^−1^ in both soils, exerted by two fungicidal treatments (Table 2). Similarly, the cellulase activity was promoted at 50 ppm by pyrazofos (as afugan) and propiconazole (as tilt) in soils inoculated with root fungi faba bean pots [41]. Captafol, at 10 parts/10^6^, was significantly inhibited mineralization of cellulose in a sandy loam soil [42]. A distinct depression was observed with chlorothalonil, under all conditions tested, that is, at the usual dose, in both flooded and nonflooded soil [43]. Similarly, trichlamide at 10 times recommended field rate (i.e., 400 mg/kg) incubated for 4 weeks under flooded soil conditions, inhibited the cellulolytic activity completely. Whereas the usual field rate the activity was about 50% that in the control soil [40]. Further Petker and Rai [44] demonstrated that five fungicides, captan, cosan, thiram, zineb, and sandolex, inhibited the cellulase activity, with greater inhibition with increasing fungicidal concentrations. According to Arinze and Yubedee [45], benlate, calixin, and captan inhibited the activity of cellulase in *Fusarium monoliforme* isolates.

## 5. Conclusion

The results obtained in the present study clearly indicate that the fungicides chlorothalonil and propiconazole profoundly enhanced the activities of both cellulase and invertase at field application rates. On the basis of these results, it is concluded that the microbial activities (i.e., enzyme activities) were increased by the fungicides applied at recommended levels in agricultural system to control insect pests.

## Figures and Tables

**Table 1:** Physico-characteristic of the soils.

Properties	Black clay soil	Red sandy loam soil
Sand (%)	68.3	53.3
Silt (%)	22.7	27.1
Clay (%)	09.0	19.6
pH^a^	7.7	6.6
Water holding capacity (mL g^−1^ soil)	0.47	0.27
Electrical conductivity (m.mhos)	254	238
Organic matter^b^ (%)	1.44	0.72
Total nitrogen^c^ (%)	0.084	0.042
NH_4_^+^-N (*μ*g g^−1^ soil)^d^	7.96	7.01
NO_2_^−^-N (*μ*g g^−1^ soil)^e^	0.48	0.32
NO_3_^−^-N (*μ*g g^−1^ soil)^f^	0.98	0.76

where, ^a^1 : 1.25 = soil : water slurry.

^b^Walkley-Black method [24].

^c^ Micro-Kjeldahl metod [24].

^d^Nesslerization method [24].

^e^Diazotization method [25].

^f^Brucine method [26].

**Table 2:** Activity of cellulase* under the impact of different concentrations of selected fungicides propiconazole and chlorothalonil in soils incubated for 24 hours after 10 days.

Concentration of fungicides (Kg ha^−1^)	Black clay soil		Red sandy loam soil	
	Propiconazole (Tilt)	Chlorothalonil (Kavach)	Propiconazole (Tilt)	Chlorothalonil (Kavach)
0.0	455 ± 2.886^a^	455 ± 2.886^a^	375 ± 5.773^a^	375 ± 5.773^a^
1.0	496 ± 5.773^a^	471 ± 11.547^a^	431 ± 17.320^b^	415 ± 8.660^b^
2.5	616 ± 3.464^b^	575 ± 5.774^b^	525 ± 2.886^c^	505 ± 1.732^b^
5.0	750 ± 11.547^c^	726 ± 1.154^c^	695 ± 2.886^d^	688 ± 1.732^c^
7.5	619 ± 1.154^d^	554 ± 2.309^d^	505 ± 2.886^c^	496 ± 1.154^b^
10.0	449 ± 0.577^a^	436 ± 4.618^a^	345 ± 2.886^e^	324 ± 1.154^d^

Each column is mean ± S.E. for six concentrations in each group; columns not sharing a common letter (a, b, c, d and e) differ significantly with each other (*P* ≤ 0.05; DMRT).

**Table:** (a)

Concentration of fungicides (Kg ha^−1^)	Propiconazole (Tilt)		Chlorothalonil (Kavach)	
	Black clay soil			
	24 hrs	48 hrs	24 hrs	48 hrs
0.0	827 ± 1.154^a^	915 ± 8.660^a^	827 ± 1.154^a^	915 ± 1.154^a^
1.0	911 ± 5.773^b^	1002 ± 1.154^b^	896 ± 2.309^b^	931 ± 17.320^b^
2.5	1152 ± 1.154^c^	1190 ± 1.154^c^	976 ± 1.732^c^	1062 ± 1.154^b^
5.0	963 ± 1.732^d^	1064 ± 1.154^d^	1183 ± 1.732^d^	1220 ± 2.309^d^
7.5	904 ± 2.309^b^	984 ± 0.577^e^	963 ± 1.732^e^	1002 ± 1.154^d^
10.0	751 ± 0.577^e^	821 ± 0.577^f^	726 ± 1.732^f^	848 ± 1.154^e^

**Table:** (b)

Concentration of fungicides (Kg ha^−1^)	Propiconazole (Tilt)		Chlorothalonil (Kavach)	
	Red sandy loam soil			
	24 hrs	48 hrs	24 hrs	48 hrs
0.0	827 ± 1.154^a^	915 ± 8.660^a^	827 ± 1.154^a^	915 ± 1.154^a^
1.0	911 ± 5.773^b^	1002 ± 1.154^b^	896 ± 2.309^b^	931 ± 17.320^b^
2.5	1152 ± 1.154^c^	1190 ± 1.154^c^	976 ± 1.732^c^	1062 ± 1.154^b^
5.0	963 ± 1.732^d^	1064 ± 1.154^d^	1183 ± 1.732^d^	1220 ± 2.309^d^
7.5	904 ± 2.309^b^	984 ± 0.577^e^	963 ± 1.732^e^	1002 ± 1.154^d^
10.0	751 ± 0.577^e^	821 ± 0.577^f^	726 ± 1.732^f^	848 ± 1.154^e^

Each column is mean ± S.E. for six concentrations in each group; columns not sharing a common letter (a, b, c, d, e, and f) differ significantly with each other (*P* ≤ 0.05; DMRT).
